# A Pan-Cancer Analysis of Age and Sex Differences in Cancer Incidence and Survival in the United States, 2001–2020

**DOI:** 10.3390/cancers17030378

**Published:** 2025-01-24

**Authors:** Rachel C. Selvaraj, Gino Cioffi, Kristin A. Waite, Sarah S. Jackson, Jill S. Barnholtz-Sloan

**Affiliations:** 1 Trans Divisional Research Program, Division of Cancer Epidemiology and Genetics, National Cancer Institute, Bethesda, MD 20892, USA; 2 Department of Statistics, University of Illinois Urbana-Champaign, Champaign, IL 61820, USA; 3 Infections and Immunoepidemiology Branch, Division of Cancer Epidemiology and Genetics, National Cancer Institute, Bethesda, MD 20892, USA; 4 Center for Biomedical Informatics and Information Technology, National Cancer Institute, Bethesda, MD 20892, USA

**Keywords:** sex differences, age, incidence, survival

## Abstract

Background: In cancer, age and sex are often studied individually, but the impact of the intersection of these factors on cancer incidence and survival remains unclear. Using population-level data, we provide an up-to-date analysis of the impact of sex and age on cancer incidence and survival. Methods: Using data from the United States Cancer Statistics public use research database and the Centers for Disease Control and Prevention’s National Program of Cancer Registries Survival database, we assessed sex and age differences in the incidence and survival of malignant cancers diagnosed from 2001 to 2020. Results: Males experienced higher cancer incidence than females in all sites and age groups, excluding 20–29- and 30–39-year-olds. The highest Male-to-female (M:F) age-adjusted incidence rates (IRR) were observed in mesothelioma within ages 80+ (IRR: 5.48; 95% CI: 5.25–5.71; *p* < 0.001), and lowest in endocrine cancer within ages 20–29 years (M:F IRR: 0.20; 95% CI: 0.20–0.21; *p* < 0.001). Among all sites and age groups, excluding 0–9 years, males experienced worse survival than females, particularly within ages 20–29 years (Hazard Ratio (HR): 2.19; 95% CI: 2.15–2.23; *p* < 0.001). Highest M:F HRs were observed in endocrine system cancers within ages 20–29 (HR: 3.52; 95% CI: 3.15–3.94; *p* < 0.001), and lowest among lymphomas within ages 0–9 (HR: 0.74; 95% CI: 0.63–0.87; *p* < 0.001). Conclusions: Significant age and sex differences in cancer incidence and survival were observed across the US from 2001 to 2020. Males had a higher cancer incidence compared to females, with notable exceptions for younger age groups among certain types, suggesting age may be a critical component in further understanding the biology of sex differences in cancer.

## Simple Summary

In cancer, age and sex are often studied individually, but the impact of the intersection of these factors on cancer incidence and survival remains unclear. Using the most up-to-date data, we provide an up-to-date analysis of the impact of sex and age on cancer incidence and survival. Using population-based data from the United States Cancer Statistics public use research database and the Centers for Disease Control and Prevention’s National Program of Cancer Registries Survival database, we assessed sex and age differences in the incidence and survival of cancers diagnosed from 2001 to 2020. Results demonstrate significant age and sex differences in cancer incidence and survival across the US from 2001 to 2020. Males had a higher cancer incidence compared to females, with notable exceptions for younger age groups among certain types, suggesting age may be a critical component in further understanding the biology of sex differences in cancer.

## 1. Introduction

Existing research identifies differences in cancer incidence and survival across both age and sex [1,2,3]. Studies are now focusing on the intersection of age and sex in various cancer types and how these variables may impact cancer incidence and survival [3]. Previous studies have shown that males generally have higher cancer incidence and mortality when compared to females [1,4,5,6]. Globally, studies have demonstrated sex differences with males having a statistically significant higher incidence of cancer compared to women [7,8]. Further, there is an observed increase in cancer incidence and mortality in older age groups [9,10]. Recent research suggests that this can be attributed to the age-associated molecular landscapes and the aging nature of cancers [9,11]. Although the causes of sex differences in cancer incidence and mortality are not yet well understood, it is becoming clear that genetic, environmental, and behavioral factors likely play a role [12,13].

We have recently shown the importance of age and sex in understanding incidence and survival in primary malignant gliomas, showing that the male-to-female incidence was lowest in those aged 0–9 years and increased with age, peaking at 50–59 years. While females diagnosed with gliomas had worse survival in those aged 0–9 years, male survival was worse in all other age groups [3]. Similarly, another study showed nuanced patterns of sex-specific incidence rates with advancing age across lymphoid neoplasm subtypes [14]. Other groups have analyzed the impact of age and sex on cancer incidence, particularly among racial and ethnic groups, in which variation in male-to-female incidence by age was observed in pancreas, colorectal, anal, and lung cancers [6].

Together, these studies suggest that there exists an interaction between age and sex. Despite this, many studies have been limited in their analysis through restricted age ranges and/or primary tumor sites. As cancer etiology can vary widely across primary sites, it is important to systematically examine age and sex differences in incidence and survival across all cancers. Identification and examination of sex differences in cancer incidence and survival can provide insight into epidemiology and etiology studies. We have previously performed a pan-cancer analysis utilizing data from the United States Cancer Statistics (USCS) public use database as well as the Surveillance, Epidemiology, and End Results (SEER) public use dataset, from 2001 to 2016, to examine the impact of sex on cancer incidence and survival [1]. Here, we update this previous study, analyzing data from 2001 to 2020 and adding the variable of age to quantify differences in cancer incidence and survival across both age and sex.

## 2. Materials and Methods

*Cancer incidence data:* Population-based cancer incidence data by primary site, sex, and age groups were queried from the USCS public use research database, which comprises data from the Centers for Disease Control and Prevention’s (CDC) National Program of Cancer Registries (NPCR) and the National Cancer Institute’s (NCI) SEER [15]. All cancer incidence data within the USCS dataset are de-identified, and cell counts with fewer than 16 cases are suppressed, as they have poor reliability. Reported data from NPCR span 46 states and the District of Columbia, and SEER accounts for 22 US geographic areas, collectively representing 99.9% of the U.S. population (cases diagnosed in Mississippi from 2001 to 2002 were not available) [16]. Data were collected from 1 January 2001 to 31 December 2020. Data analysis took place from 12 June to 2 August 2024.

*Survival data:* Survival data by age, sex, and primary site were obtained from the NPCR survival database, which consists of 43 central cancer registries, covering 91.7% of the total US population [17]. Statistics based on fewer than 16 cases were suppressed.

*Selection criteria:* Cases were classified according to the International Classification of Diseases for Oncology, Third Edition (ICD-O-3) [18]. Primary site groupings were classified by the site recode ICD-O-3/WHO 2008 definition provided in the UCSC SEER*Stat database, with only malignant behavior codes (ICD-O-3 behavior code of 3) being considered for analysis [19]. Only microscopically or radiographically confirmed cases were considered in this study. Data were restricted to patients with first-primary tumors. Sex-specific cancers (e.g., cervical, ovarian, and prostate), and cancers with a notably large predisposition for a particular sex (e.g., breast cancer for females and Kaposi sarcoma for males) were excluded from this analysis, due to inherent sex differences.

*Statistical analysis:* Using age at diagnosis, 10-year age intervals were used to create the following age groups: 0–9, 10–19, 20–29, 30–39, 40–49, 50–59, 60–69, 70–79, and 80+ years. These intervals were selected as they allowed for a more granular assessment across the age spectrum while also providing sufficient sample sizes for analysis. Male-to-female (M:F) incidence rate ratios (IRRs) with 95% confidence intervals (95% CI) were generated for all age groups and primary sites to compare incidence across sexes. All rates were age-adjusted to the 2000 U.S. standard population (19 age groups–Census P25-1130) to account for differences in age distribution by sex [20]. Survival time was calculated as the number of months from diagnosis to death. Age-stratified Cox proportional hazard models were constructed to calculate M:F hazard ratios HRs) with 95% CIs. The Cox proportional hazards assumption was assessed by generating log-log plots, and models were not found to be in violation. All cases assessed were complete; there were no data removed for missingness. Cases that were suppressed in cancer-specific analyses are included in “All Sites”. Statistical significance was set at *p* < 0.05 and all reported *p* values are two-tailed. Incidence calculations were generated using SEER*Stat 8.4.3. Survival analyses were performed through R (version 4.4.0) using the R package “survival”. All plots were generated through R (version 4.4.0) using the R package “ggplot2”.

## 3. Results

A total of 15,769,051 individuals [8,823,617 male (56%) and 6,945,434 female (44%)] received a primary cancer diagnosis of a non-sex-specific malignancy from 2001 to 2020. Survival analyses were conducted on 14,462,616 individuals with a non-sex-specific type of cancer [8,090,970 males (56%) and 6,371,646 females (44%)].

**Figure 1:**
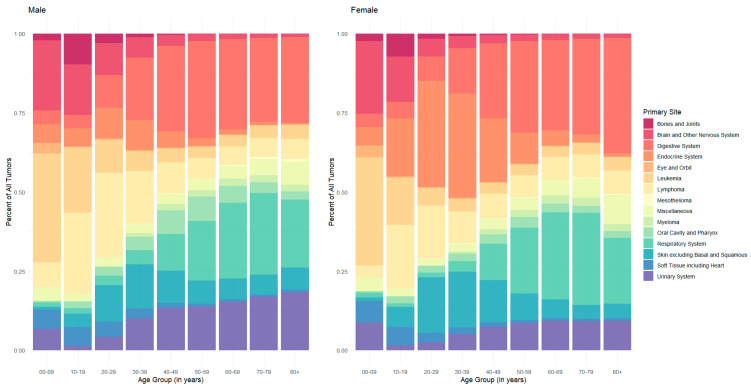
Frequency and percent of all tumors by sex, age in years, and SEER Site Recode ICD-O-3/WHO 2008 major groupings. National Program of Cancer Registries and Surveillance, Epidemiology and End Results Program SEER*Stat Database: NPCR and SEER Incidence—U.S. Cancer Statistics Public Use Research Database, 2023 Submission (2001–2020).

In general, males had an overall observed higher cancer incidence at all sites and age groups. The exception to this was in those aged 20–29 years and 30–39 years, (Supplementary Table S1). Females who were either 20–29 years or 30–39 years had a higher incidence across all primary sites than males within the same age group (M:F IRR: 0.71; 95% CI: 0.70–0.71; *p* < 0.001; M:F IRR: 0.81; 95% CI: 0.80–0.81; *p* < 0.001, respectively). Incidence across all primary sites varied widely between both age and sex (Figure 1). For example, endocrine system cancers accounted for 33% of all tumors for females aged 20–29 years and 30–39 years (N = 54,732, N = 104,852, respectively) (Figure 1). In contrast, endocrine system cancers accounted for 10% of all tumors for males aged 20–29 years (N = 11,530) and 9% for males aged 30–39 years (N = 24,226) (Figure 1).

**Figure 2:**
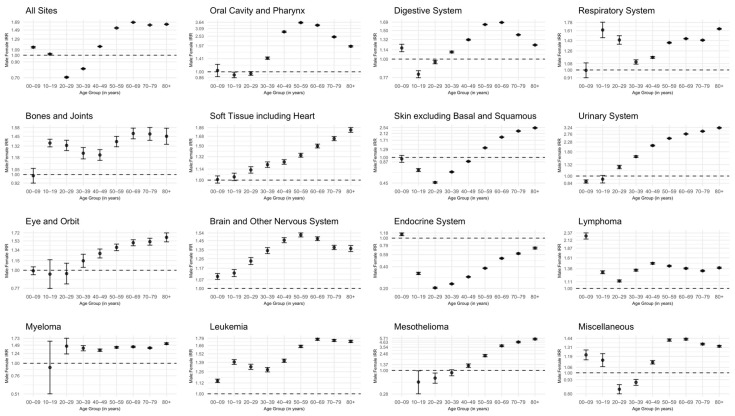
Forest plots of incidence rate ratios (IRRs) by sex (Male:Female), age in years, and SEER Site Recode ICD-O-3/WHO 2008 major groupings with 95% confidence intervals. National Program of Cancer Registries and Surveillance, Epidemiology and End Results Program SEER*Stat Database: NPCR and SEER Incidence—U.S. Cancer Statistics Public Use Research Database, 2023 Submission (2001–2020). Rates are per 100,000 and age-adjusted to the 2000 U.S. standard population.

Highest M:F IRRs were seen in those diagnosed with mesothelioma at an older age (60–69 years M:F IRR: 3.79; 95% CI: 3.63–3.97; *p* < 0.001; 70–79 years M:F IRR: 4.66; 95% CI: 4.48–4.84; *p* < 0.001; 80+ years M:F IRR: 5.48; 95% CI: 5.25–5.71; *p* < 0.001) (Figure 2). Endocrine cancer, in those 20–29 years, had the lowest M:F IRRs were lowest (M:F IRR: 0.20; 95% CI: 0.20–0.21; *p* < 0.001) (Figure 2). A higher incidence of oral cavity and pharynx cancers was found in females aged 10–19 years and 20–29 years (M:F IRR: 0.92; 95% CI: 0.86–0.99; *p* = 0.029; M:F IRR: 0.96; 95% CI: 0.91–1.00; *p* = 0.078, respectively) (Figure 2). In general, males had a higher incidence of digestive system cancer. Digestive system cancers were higher in males. The exception to this was in those aged 10–19 years and 20–29 years (M:F IRR: 0.81; 95% CI: 0.77–0.85; *p* < 0.001, M:F IRR: 0.96; 95% CI: 0.94–0.98; *p* < 0.001, respectively) (Figure 2). For skin cancer, excluding basal and squamous cell skin cancer, M:F IRRs were highest within those aged 70–79) and 80+ (M:F IRR: 2.28; 95% CI: 2.26–2.30; *p* < 0.001; M:F IRR: 2.52; 95% CI: 2.49–2.54; *p* < 0.001, respectively) (Figure 2). Soft tissue cancers had the highest M:F IRRs, specifically among those aged 70–79 and 80+ (M:F IRR: 1.6; 95% CI: 1.59–1.68; *p* < 0.001; M:F IRR: 1.81; 95% CI: 1.76–1.86; *p* < 0.001, respectively) (Figure 2). When analyzing those age 70–79 and 80+ years, eye and orbit cancers had the highest observed M:F IRRs (M:F IRR: 1.51; 95% CI: 1.44–1.59; *p* < 0.0001, M:F IRR: 1.61; 95% CI: 1.51–1.72; *p* < 0.0001, respectively). M:F IRRs of urinary system cancers increased with age, maxing at ages 80+ (M:F IRR: 3.21; 95% CI: 3.19–3.24; *p* < 0.001) (Figure 2). Brain and other nervous systems cancers, leukemias, and lymphomas exhibited higher incidence in males across all age groups (*p* < 0.001) (Figure 2).

**Figure 3:**
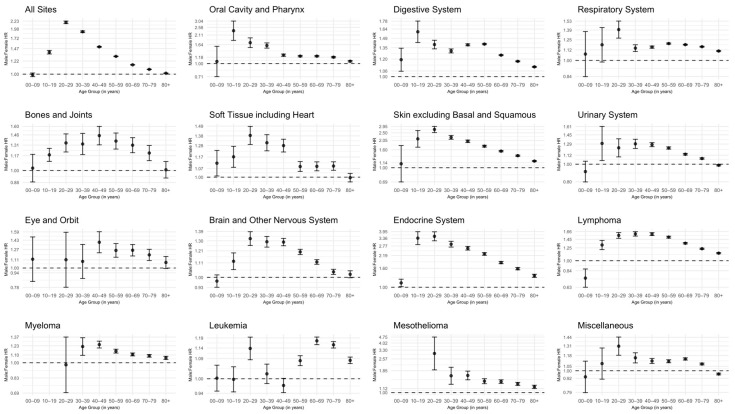
Forest plot of male-to-female hazard ratios (HRs) by age in years and SEER Site Recode ICD-O-3/WHO 2008 major groupings with 95% confidence intervals. NPCR survival database, November 2023 Submission, Diagnosis Year 2001–2020.

M:F HRs were calculated by age group for all sites (Figure 3, Supplementary Table S2). Overall, males experienced worse survival compared to females for all sites and age groups, excluding those aged 0–9 years (*p* < 0.001) (Figure 3). This was particularly evident in the 20–29 year age group (M:F HR: 2.19; 95% CI: 2.15–2.23; *p* < 0.001) (Figure 3). Endocrine system cancers, in those aged 10–19 and 20–29 years had the highest M:F HRs (M:R HR: 3.37; 95% CI: 2.87–3.95; *p* < 0.001; M:F HR: 3.52; 95% CI: 3.15–3.94; *p* < 0.001, respectively) (Figure 3). Compared to males, females observed the worst survival for lymphomas within those aged 0–9 years (M:F HR: 0.74; 95% CI: 0.63–0.87; *p* < 0.001). In contrast, males experienced worse survival than all other age groups (*p* < 0.001) (Figure 3). In general, in those diagnosed with mesothelioma, males had worse survival compared to females (*p* < 0.001), particularly in those aged 20–29 years (M:F HR: 3.00; 95% CI: 1.89–4.75; *p* < 0.001) (Figure 3). This was not seen in those 0–9 and 10–19 years old. For oral cavity and pharynx cancers, males experienced worse survival when compared to females for all age groups (*p* < 0.001), except those aged 0–9 years (*p* = 0.782). The highest HRs for oral cavity and pharynx cancer were observed in those aged 10–19 and 20–29 years (M:F HR: 2.36; 95% CI: 1.84–3.04; *p* < 0.001; M:F HR: 1.73; 95% CI: 1.52–1.96; *p* < 0.001) (Figure 3). For cancers of the skin, excluding basal and squamous, those aged 20–29 years had the highest HR (M:F HR: 2.73; 95% CI: 2.52–2.95; *p* < 0.001) (Figure 3).

## 4. Discussion

Consistent with previous work looking at specific tumor sites and different datasets [1,4,5,6,9,10], this update of our previous pan-cancer analysis identified significant age and sex differences in cancer incidence across the US from 2001 to 2020. Overall, males had higher cancer incidence compared to females, consistent with existing literature [1,21,22]. However, notable exceptions existed within specific age groups as well as within specific cancer sites/types. Specifically, females between the ages of 20–29 years and 30–39 years exhibited a higher incidence compared to males within the same respective age groups, driven heavily by endocrine system cancers. Past research suggests that a higher incidence of endocrine system cancers in females may be attributed to hormonal factors and reproductive history [23,24]. Additionally, LeClair and colleagues have shown that females are more likely to have smaller, subclinical papillary thyroid cancer, identified through medical testing compared to men [25] and this may be a contributing factor in the differences observed here. In contrast to what was observed with endocrine tumor incidence, males exhibited significantly higher incidence rates for mesotheliomas and digestive system cancers, particularly in older age groups when compared to females. This is not surprising as evidence suggests that occupational exposures and behavioral factors, such as smoking and diet, play a large role in these sex differences [26,27,28,29]. Females may have better incidence rates, compared to males, due to the possible protective factors of sex hormones, tumor suppressor genes on the X chromosomes, and social behaviors.

Regarding cancer survival, and in support of previous studies [4,30,31], males generally had worse outcomes compared to females across most cancer types and age groups, with few exceptions. Among all sites, except within ages 20–29 years males exhibited worse survival outcomes compared to females [4,30,31]. Interestingly, while males had a lower incidence of endocrine tumors, males had a worse survival outcome compared to females. It may be interesting to postulate that this sex difference in survival may be due to differences in histopathological types or stages at diagnosis of endocrine tumors in males and females. The highest survival differences were observed in mesothelioma and endocrine system cancers particularly within persons aged 20–29 years. In addition, across most sites, M:F HRs decreased as the age group increased, implying that the magnitude of sex differences in survival may decrease as patients age. Some studies suggest that biological differences attributed to age, such as variations in immunosenescence, might contribute to the observed differences [32,33].

As touched upon above, observed age and sex differences in cancer incidence and survival are likely multifactorial, involving a combination of biological, environmental, and behavioral factors. Recent work examining cancer incidence among generations [34] combined with other studies examining changes in cancer registry policies [35] as well as increased imaging practices [36,37,38,39] suggests that the increased incidence rate of cancer in Generation X is undoubtedly complicated. While this study does not examine age in the same manner, these recent works highlight the need to remain cognizant of the potential impact of behavioral factors that may impact reported incidence levels. Biological factors that may be influential include hormonal differences and variations in immune system development [40]. Females face unique biological factors, including reproductive factors and sex hormones, that contribute to cancer incidence. It has been well established that sex hormones play a critical role in the development and progression of cancer in females. For instance, the protective effects of estrogen in females might partly explain the lower incidence and better survival rates across many cancer sites [41]. While these studies were originally conducted in reproductive cancers, continuing research has shown that these hormones also play an important role in non-reproductive cancers. Studies have demonstrated that estrogen and progesterone, and their respective receptors have an important role in cellular signaling for cellular growth and metastasis [42,43,44,45,46]. In addition, growing evidence also suggests that membrane receptors mediate non-classical actions [45,47]. Activation of estrogen receptors stimulates cellular proliferation and differentiation [44,48,49]. Progesterone, besides having its own growth mechanism, can also modulate the actions of estrogen, increasing proliferation and the potential for cancer development and progression [43,47]. Lastly, sex hormones have also been implicated in regulating apoptosis and cellular suppression [50,51,52,53,54,55]. The understanding of how sex hormones contribute to cancer in women continues to drive the development of targeted prevention and therapies [41]. The interplay between these factors is complex and is the focus of active research.

Environmental exposures, such as occupational hazards and behavioral factors may also heavily influence the observed differences. Further, many social behaviors have been linked to impacting cancer risk. These include tobacco use, alcohol consumption, diet, and physical activity. Historically, men have smoked more than women; however, the smoking gap has closed between the sexes, which has correlated to a shrinking of the risk gap between men and women [56,57]. Some studies attribute higher incidence rates of mesotheliomas in males to increased asbestos exposure [58,59]. Like tobacco usage, alcohol consumption is also considered a risk factor for cancers and impacts men to a greater extent due to historically higher consumption in men compared to women. Alcohol consumption increases chronic inflammation [60,61,62,63] and oxidative stress [64,65,66,67] both of which have been implicated in the development and progression of cancer. Increased alcohol consumption among older males may lead to higher incidence rates of urinary system and digestive system cancers, consistent with the findings of this study [21,68,69]. Changes in lifestyle factors, such as increased sedentary behaviors, have resulted globally, in a steady increase in average body weight and obesity [70]. Obesity is considered a major unrecognized factor for cancer, resulting in calls for action from health providers including the American Society of Clinical Oncology [71]. Numerous studies have linked obesity and diet to increased risk of cancer, though specific dietary nutrients or components have not been definitively identified. Physical inactivity and higher weight often go together and are well-documented risk factors for obesity-associated cancers. Historically, men have increased physical activity compared to women due to recreational and occupational activity [72]. Behavioral factors, such as health-seeking behaviors and the utilization of healthcare services, might further contribute to these differences, as there are different male and female risk factors that are involved in implementing risk screenings [73]. Additionally, social inequities have been demonstrated based on sex, as well as other factors. Undoubtedly, there is a complex interaction between the variety of SDOH components and sex and further analysis of these factors is warranted in order to reduce barriers to access and treatment. Past studies identify middle-aged females as exhibiting better survival outcomes than middle-aged males due to females generally exhibiting more proactive health behaviors compared to males [74,75]. While it is unlikely that just one of these behavioral factors is the sole cause of the results seen here, combined effects may contribute to the sex and age differences observed and it will be important to consider both sex and age in future studies assessing these factors. Additionally, sex differences may still be present even after accounting for these factors, so the complete cause is still largely unknown [21].

While the cancer registry data used in the analysis currently represent the most complete dataset for cancer incidence and survival in the US, there are several limitations in this study’s design and statistical analysis. The variables available for consideration in the analysis were limited to only those available through cancer registry datasets. As such, some additional variables that may influence age and sex differences in cancer incidence and survival (i.e., treatment history, pathogenesis, molecular biomarkers, socioeconomic status, etc.) were not included in the analysis. Furthermore, potential variations in findings due to race, region, stage, and treatment were not adjusted for or considered in the analysis as it is outside the scope of this current work. Survival analyses incorporating treatment, such as surgery, and stage at diagnosis would be better suited for analyses focused on specific cancer sites.

## 5. Conclusions

Identification and examination of sex differences in cancer incidence and survival can provide insight for future epidemiology and etiology studies as well as provide important points of reference when evaluating changes in practices. Future areas of research may seek to integrate more comprehensive data, including the variables noted above, to better explore the underlying causes of age and sex differences in cancer incidence and survival. The findings of this study may motivate future longitudinal studies focusing on the intersection between biological, environmental, and behavioral factors, which may inform targeted interventions and improve cancer outcomes for males and females across all age groups.

## Data Availability

USCS Incidence Data are publicly accessible and can be requested at the following website: https://www.cdc.gov/united-states-cancer-statistics/public-use/access-data.html (accessed on 20 July 2024). Data used for survival analyses were obtained from the Centers for Disease Control and Prevention’s (CDC) NPCR survival database.

## Supplementary Materials

The following supporting information can be downloaded at: https://www.mdpi.com/article/10.3390/cancers17030378/s1, Table S1: Frequency and Incidence Rate Ratios by Sex and Age Groups in SEER Site Recode ICD-O-3/WHO 2008 Sites, 2001–2020, National Program of Cancer Registries and Surveillance, Epidemiology, and End Results SEER*Stat Database: NPCR and SEER Incidence—U.S. Cancer Statistics Public Use Research Database, Nov 2023 Submission. Table S2. Hazard Ratios by Sex (Males:Females) and Age Groups with 95% Confidence Intervals in SEER Site Recode ICD-O-3/WHO 2008 Sites, 2001–2020, National Program of Cancer Registries Survival Database, Nov 2023 Submission.
